# Black Pepper (*Piper nigrum*) Alleviates Oxidative Stress, Exerts Potential Anti-Glycation and Anti-AChE Activity: A Multitargeting Neuroprotective Agent against Neurodegenerative Diseases

**DOI:** 10.3390/antiox12051089

**Published:** 2023-05-12

**Authors:** Himadri Sharma, Niti Sharma, Seong Soo A. An

**Affiliations:** Department of Bionano Technology, Gachon Bionano Research Institute, Gachon University, 1342 Seongnam-daero, Sujung-gu, Seongnam-si 461-701, Gyeonggi-do, Republic of Korea

**Keywords:** neuroprotection, *Piper nigrum*, H_2_O_2_-induced stress, anti-acetylcholine esterase activity, anti-glycation, oxidative stress

## Abstract

Neurodegenerative diseases (NDs) are a family of disorders that cause progressive structural and functional degeneration of neurons. Among all the organs in the body, the brain is the one that is the most affected by the production and accumulation of ROS. Various studies have shown that an increase in oxidative stress is a common pathophysiology for almost all NDs, which further affects various other pathways. The available drugs lack the wide spectrum necessary to confront these complexities altogether. Hence, a safe therapeutic approach to target multiple pathways is highly desirable. In the present study, the hexane and ethyl acetate extracts of *Piper nigrum* (black pepper), an important spice, were evaluated for their neuroprotective potential in hydrogen peroxide-induced oxidative stress in human neuroblastoma cells (SH-SY5Y). The extracts were also subjected to GC/MS to identify the important bioactives present. The extracts exhibited neuroprotection by significantly decreasing the oxidative stress and restoring the mitochondrial membrane potential in the cells. Additionally, the extracts displayed potent anti-glycation and significant anti-Aβ fibrilization activities. The extracts were competitive inhibitors of AChE. The multitarget neuroprotective mechanism displayed by *Piper nigrum* indicates it as a potential candidate in the treatment of NDs.

## 1. Introduction

Neurodegenerative diseases (NDs) are a family of disorders (Alzheimer’s disease, Amyotrophic lateral sclerosis, Huntington’s disease, Multiple sclerosis, Parkinson’s disease, Prion disease, etc.) that lead to the progressive structural and/or functional degeneration of neurons. ND pathophysiology involves neuronal malfunction, synaptic dysfunction, and aggregation of specific proteins in the brain [1]. The progression, region affected, and extent of neurodegeneration in the brain are variable in different types of NDs. An increase in oxidative stress is a key point in defining the etiology of neurodegeneration. Oxygen is essential for the cells to meet their energetic demands, but the consumption of oxygen can also result in free radical production which can result in cellular damage [2]. Its accumulation may induce all the factors that are responsible for aging and the development of neurological disorders, such as cellular damage, mitochondrial cell death, and impairment of the DNA repair system. In addition, oxidative stress can be generated in the brain as a result of some environmental toxin or chemical which can produce ROS as a by-product [3]. This validates the need to screen new and safe medicinal agents from natural resources. Continuous efforts are being made to find agents that can lower oxidative stress in cells and provide protection from the risk of developing neurodegenerative disorders [3]. In this perspective, numerous plants/bioactives have been reported to inhibit the production of free radicals [4] and provide neuroprotection.

Black pepper (*Piper nigrum* L.; Piperaceae family) is a flowering vine native to South Asia, crowned as the “King of Spices” due to its significant place culinarily for over 2000 years, and was the most valuable spice (called “Black Gold”) traded from India to different parts of the world. The peppercorns were used as currency by ancient Greeks and European countries between 500 and 1500 AD [5]. It is traditionally used in the treatment of colds/coughs, neuropathic pain, gastric discomfort, respiratory diseases, etc. [6] and it has been reported to have numerous pharmacological actions, viz., antioxidant, anticancer, anti-asthmatic, antihypertensive, anti-inflammatory, anti-obesity, analgesic, CNS stimulant, hepatoprotective, immuno-modulatory, and antimicrobial properties [7]. The main bioactive component of black pepper is the alkaloid piperine which has been reported to have anti-AChE and anti-amyloid activity [8,9] and can restore the levels of antioxidant enzymes [10]. Additionally, due to its antioxidant properties, it has been shown to protect against cognitive decline and hippocampal nerve damage [11] and improve long-term potentiation (LTP) in the synaptic plasticity-impaired rat model [12].

Therefore, based on the above therapeutic importance of black pepper, we explored the role of the hexane and ethyl acetate extracts of black pepper (dried fruit) on H_2_O_2_-induced oxidative stress in SH-SY5Y neuronal cells. Moreover, the effect of black pepper extract on other important targets of neurodegeneration was also evaluated by studying its anti-fibrillation activity, acetylcholine esterase (AChE) inhibition, and advanced glycation end product (AGE) inhibition.

## 2. Materials and Methods

### 2.1. Chemicals

The chemicals 6,6′-dinitro-3,3′-dithiodibenzoic acid, bis(3-carboxy-4-nitrophenyl) disulfide (DTNB), acetyl thiocholine chloride, galantamine, gallic acid, ascorbic acid, 2,2′-azinobis-(3-ethylbenzothiazoline-6-sulfonic acid) (ABTS), 2,2-diphenyl-1-picrylhydrazyl (DPPH), 2,4,6-tripyridyl-s-triazine (TPTZ), Folin–Ciocalteu reagent (FCR), 2′,7′-dichlorofluorescin diacetate (DCFDA), tetramethylrhodamine, ethyl ester (TMRE), hydrogen peroxide, thioflavin T (ThT), bovine serum albumin (BSA), sodium azide, aminoguanidine, dextrose, and acetylcholinesterase (Electrophorus electricus, Type VI-S) were bought from Sigma-Aldrich (St. Louis, MO, USA). Aβ1-42 (Aggresure™) was acquired from AnaSpec (Fremont, CA, USA). The WST-8 kit was obtained from Roche Diagnostics GmbH (Mannheim, Germany). All organic solvents of HPLC grade were purchased from Sigma-Aldrich.

### 2.2. Plant Material and Extraction

The dried black pepper fruits were procured from the Expat Mart (Seoul, Republic of Korea). The samples were weighed and powdered using a pestle mortar. The powder was extracted sequentially in n-hexane and ethyl acetate. The extracted fractions were dried, weighed, and stored at 4 °C until further experiments. 

### 2.3. Gas Chromatography–Mass Spectrometry (GC–MS) Method

The sample was analyzed on a fused-silica capillary column (DB-5ms UI, 30 m × 0.25 mm i.d., film thickness 0.25 μm, Agilent, Santa Clara, CA, USA) installed on a GCMS-QP2020 (Shimadzu, Kyoto, Japan). The oven temperature was programmed at 60 °C for 2 min, 100 °C at 4 °C/min, 290 °C at 10 °C/min, and finally to isothermic for 10 min. The split injection mode (1:10) was used and hexane and ethyl acetate fractions (1 μL, 1 mg/mL) were injected into the GC/MS via an auto-injector. The carrier gas was helium at a constant flow mode rate of 1 mL/min. The injection port, ion source, and interface temperatures were: 280, 280, and 150 °C, respectively. The energy of ionization was 70 eV. The mass spectra were obtained in full scan mode (40–700 AMU). 

### 2.4. Determination of Total Phenolic Content

The Folin−Ciocalteu method [13], with modification for the 96-well format, was used to determine the total phenolic content of the extracts. Briefly, the extracts were incubated with 1N Folin−Ciocalteu reagent for 5 min at room temperature (RT) followed by the addition of a 10% sodium carbonate solution. The 96-well plate was incubated in the dark for 2 h at RT and the absorbance was measured at 765 nm (Multimode reader, Synergy-H1 BioTek, Agilent, Santa Clara, CA, USA). Gallic acid (10–200 mg/L) was used as a standard for calibration, and the results are expressed as mg gallic acid equivalent (GAE)/g of extract.

### 2.5. Determination of Total Flavonoids Content

The total flavonoids were estimated following the method of Ribarova et al. [14], with modifications for the 96-well plate format. To the extract, 10% aluminum chloride, 96% ethanol, and 1M sodium acetate were added and the 96-well plate was incubated in the dark at RT for 40 min. The absorbance was measured at 415 nm using a microplate reader (Synergy-H1 BioTek, Agilent, Santa Clara, CA, USA). The quercetin standard curve (10–100 µg/mL) was used to estimate the flavonoids in the extract and the results were expressed as mg quercetin equivalents per gram of sample (mg/g).

### 2.6. Determination of Antioxidant Capacity

#### 2.6.1. Free Radical Scavenging by 2,2-Diphenyl-1-picrylhydrazylhydrate (DPPH) Radical

The method described previously [15], with minor modifications, was used to determine the DPPH radical scavenging capacity of the extracts. The diluted extract was mixed with 120 µM ethanolic DPPH. The 96-well plate was incubated in the dark for 30 min at RT and the absorbance was checked at 515 nm (Multimode reader, Synergy-H1 BioTek, Agilent, Santa Clara, CA, USA). Ascorbic acid (0.1–10 µg/mL) was used as a standard. Radical scavenging activity (RSA) was calculated using the following formula:

% RSA = (Ab − Ae/Ab) × 100

where Ab = absorbance of the blank and Ae = absorbance of the extract.

#### 2.6.2. Free Radical Scavenging by 2,2′-Azino-bis (3-Ethylbenzothiazoline-6-Sulfonic Acid) [ABTS] Radical

The free radical scavenging capacity of extracts was measured by the method described earlier [16]. ABTS radicals were generated by mixing equal volumes of ABTS (0.7 mM) and potassium persulfate (2.45 mM) kept in the dark at RT for 30 min. The extract was mixed with the ABTS radical solution and incubated in the dark for 30 min at RT. The absorbance was measured at 734 nm using the microplate reader (Synergy-H1 BioTek, Agilent, USA). Ascorbic acid (100 µg/mL) was used as a standard. The percentage of inhibition of ABTS+• was calculated as:

% RSA = (Ab − Ae/Ab) × 100

where Ab = absorbance of the blank and Ae = absorbance of the extract.

#### 2.6.3. Ferric Reducing Antioxidant Potential (FRAP) Assay

The FRAP assay was carried out to evaluate the metal-chelating ability of the extracts by modifying a previously described method [17]. The working FRAP reagent was prepared by mixing 10:1:1 volumes of 300 mM acetate buffer (pH 3.6), 10 mM TPTZ (2,4,6-tri(2-pyridyl)-s-triazine) in 40 mM hydrochloric acid, and 20 mM ferric chloride. A standard curve was prepared using FeSO_4_·7H_2_O at various concentrations (1 mM). For the assay, the extract was incubated with 300 µL of FRAP reagent, and the reduction of ferric tripyridyltriazine to a ferrous complex by the extract was monitored at 593 nm (Multimode reader, Synergy-H1 BioTek, Agilent, USA) after 30 min of incubation at RT. FRAP values of the sample were expressed as µM Fe^2+^/g. 

### 2.7. Acetylcholinesterase Inhibitory Activity

The AChE activity was monitored by slight modifications to Ellman’s method [18]. The extracts were incubated at 37 °C for 15 min with AChE and 10 mM ATCC in a phosphate buffer (100 mM, pH 7.6). The reaction was terminated by 15 mM DTNB, and the absorbance was measured at 412 nm using the plate reader (Synergy-H1 BioTek, Agilent, USA). Galantamine served as a positive control. The percent inhibition was calculated as:

Percent Inhibitory activity (I%) = [(Ao − Ac) − (Bi − Bc)]/(Ao − Ac) × 100

where Ao is the absorbance without inhibitor; Ac is the negative control without inhibitor; Bi is the absorbance with inhibitor; and Bc is the negative control with inhibitor. The IC_50_ values were determined by GraphPad Prism 9.5. 

### 2.8. Thioflavin T (ThT) Assay

A previously reported method was followed with slight modification [19]. The assay was performed using 5 μM Aβ1-42 (Aggresure™ AnaSpec) in PBS (100 mM, pH 7.4) and was incubated with or without extract for 24 h at 37 °C. Next, 100 μM ThT was added, and the plate was incubated for 15 min more at 37 °C; after which the fluorescence (Ex 450 nm; Ems 490 nm) was measured (Synergy-H1 BioTek, Agilent, USA). Phenol red (100 μM) was used as the inhibitor control. 

The aggregation inhibition was calculated as:

Inhibition (%) = (Fc − Fi)/Fc × 100%

where Fi and Fc are the fluorescence intensity with and without the inhibitors, respectively. 

### 2.9. Advanced Glycation End-Product (AGE) Inhibition Activity

The glycation reaction was carried out as described [20] by incubating the extracts in 100 mM phosphate buffer (pH 7.4) containing BSA, dextrose monohydrate, and sodium azide at 37 °C for 14 days. Aminoguanidine was used as a positive control. The fluorescence (Ex 370 nm; Ems 440 nm) was measured (Multimode reader, Synergy-H1 BioTek, Agilent, USA) and the percent glycation inhibition was calculated as:

Inhibition (%) = [(C − T)/C  ×  100]

where C and T are the fluorescence intensity in the absence and presence of the sample, respectively. The IC_50_ values were determined by GraphPad Prism 9.5.

### 2.10. Cell Culture 

Human neuroblastoma SH-SY5Y cells (ATCC CRL-2266, Manassas, VA, USA) were maintained in Dulbecco’s modified Eagle’s medium (DMEM, Gibco) supplemented with 10% fetal bovine serum (FBS), 1% kanamycin, and 1% penicillin (Thermo Fisher Scientific, Waltham, MA, USA) at 37 °C with 5% CO_2_, and a 95% humidified atmosphere in the incubator. The cells were passaged twice per week and the experiments were performed at 80–90% cell confluency.

#### 2.10.1. Cell Viability Assay

For the cell viability assay, cells were seeded (1 × 10^4^ cells/well) in sterile 96-well plates and subjected to various concentrations of extracts for 24 h. The extracts were removed and the cells were washed twice with 1X PBS and incubated in the fresh medium with 10% WST-8 reagent (Roche, Grenzach-Wyhlen, Germany) for 2 h. The absorbance was measured at 450 nm in a multi-plate reader (Synergy-H1 BioTek, Agilent, USA). The percent cytotoxicity was calculated as:

Cytotoxicity % = (A control cells − A treated cells)/(A control cells) × 100

where A control cells = absorbance of the control cells and A treated cells = absorbance of the treated cells.

The plot of percent cytotoxicity versus sample concentration was used to calculate the extract concentration that killed 50% of the cells (IC_50_).

#### 2.10.2. Neuroprotective Activity Assay 

The neuroprotective effect of extracts on H_2_O_2_-induced oxidative stress in SH-SY5Y was performed as previously described [19]. The cells (1 × 10^4^ cells/well) were seeded in a 96-well plate and incubated for 18–24 h. After stabilization, the cells were pre-treated with the extracts for 24 h. The extracts were then removed and treated with H_2_O_2_ (100 μM) for 6 h. A solvent control, H_2_O_2_ alone, and extract alone treatments were also included. After incubation, the % cell viability was determined using WST-8 reagent in triplicate experiments. 

#### 2.10.3. Measurement of Intracellular Reactive Oxygen Species (ROS) 

The ROS was measured using 2′,7′-dichlorodihydrofluorescein diacetate (H2DCFDA) as previously described [19]. The cells (1 × 10^4^ cells/well) were seeded in a 96-well plate and incubated for 18–24 h, after which they were pre-treated with the extract for 12 h. The extracts were then removed, followed by a 4 h treatment with H_2_O_2_ (100 μM). Then, 25 μM H2DCFDA was added and the cells were incubated for another 2 h in the dark at 37 °C. The fluorescence intensity (Ex 495 nm, Ems 520 nm) was measured by a microplate reader (Synergy-H1 BioTek, Agilent, USA). The ROS was calculated as a percentage of the untreated control cells (100%) in triplicate measurements.

#### 2.10.4. Mitochondrial Membrane Potential (ΔΨm) Assay 

The mitochondrial membrane potential was measured using the tetramethylrhodamine, methyl ester (TMRE) staining method [21]. The cells (1 × 10^4^ cells/well) were seeded in a 96-well plate and incubated for 18–24 h; after which, they were pre-treated with the extract for 12 h. The extracts were then removed, followed by a 2 h treatment with H_2_O_2_ (200 μM). The cells were incubated for 1 h with 1 μM TMRE at 37 °C. The fluorescence (Ex 549 nm, Ems 575 nm) was read in a microplate reader (Synergy-H1 BioTek, Agilent, USA). The ΔΨm was calculated as a percentage of the untreated control cells (100%) in triplicate measurements.

#### 2.10.5. Statistical Analysis 

Statistical analysis was established by a one-way ANOVA followed by Dunnett’s post-hoc test. Data are registered as the mean ± SD of at least three experiments. The symbols ###, *** represents *p* < 0.001, ##, ** represents *p* < 0.01, and #, * represents *p* < 0.05. The symbol # indicates significance compared to the H_2_O_2_ control while * indicates significance compared to the untreated control. The IC_50_ values were determined using non-linear regression. The Michaelis–Menten plot was drawn using a non-linear plot by GraphPad Prism 9.5, and the Vmax and Km were calculated from it. Lineweaver–Burk plots were drawn using linear regression analysis by GraphPad Prism 9.5. 

## 3. Results and Discussion

### 3.1. GC–MS Analysis

**Figure 1:**
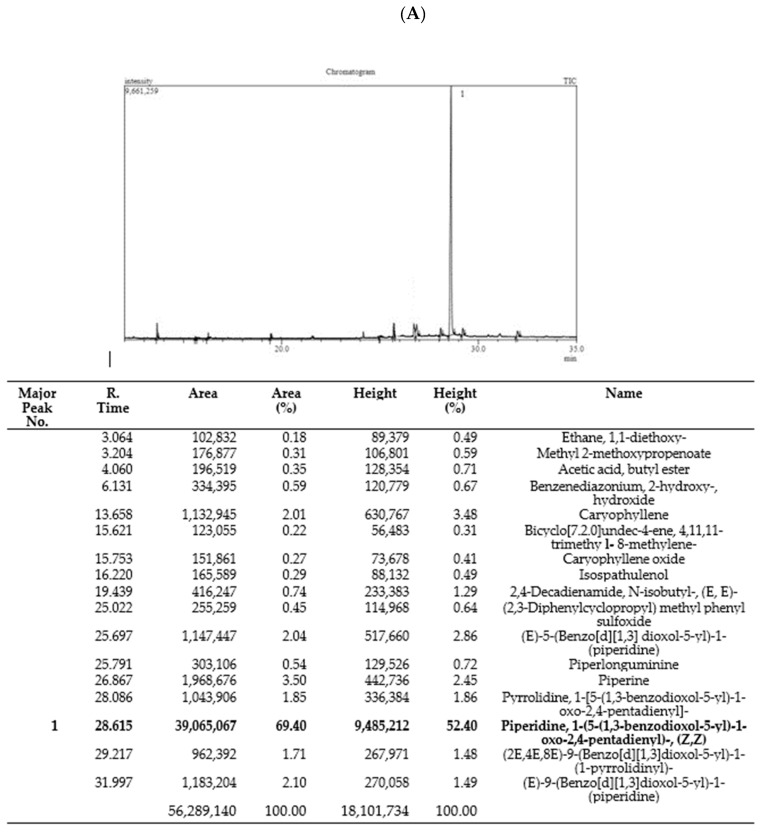

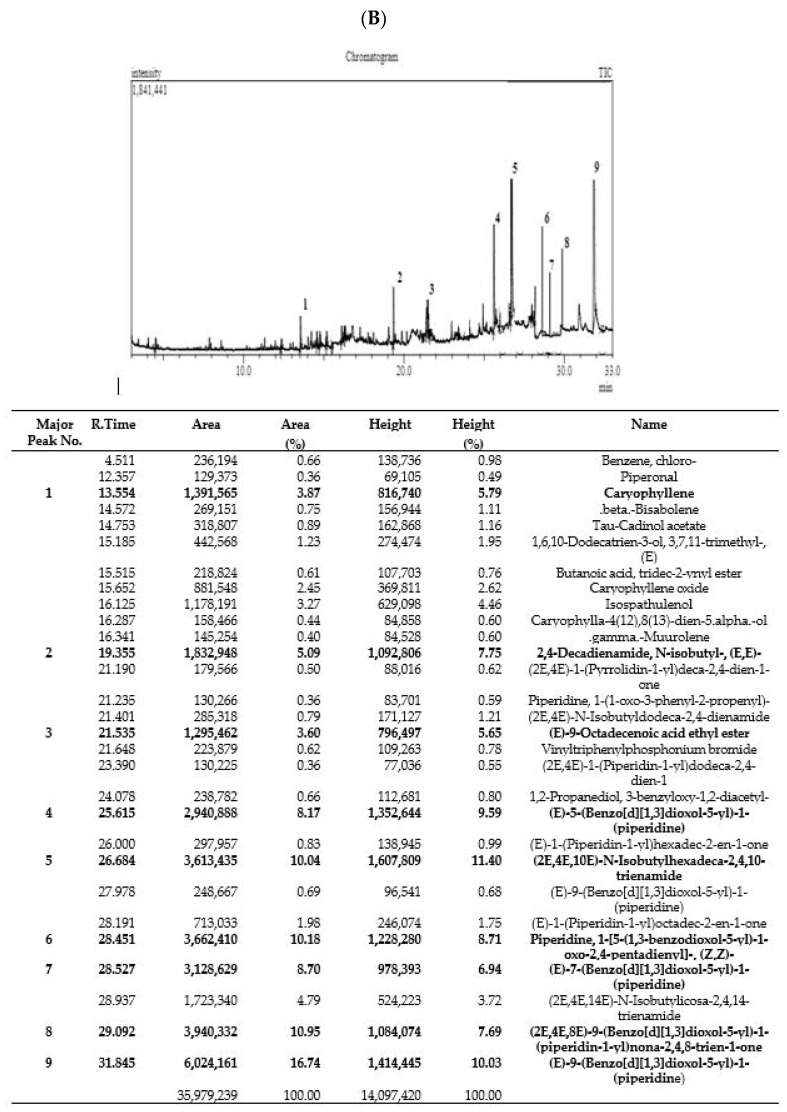
Phytoconstituents identified in Pep-EA (**A**) and Pep-H (**B**) extracts from *Piper nigrum* using gas chromatography–mass spectrometry. Abbreviation: Pep-EA: Pepper-Ethyl acetate; Pep-H: Pepper-Hexane.

The GC–MS chromatogram of *Piper nigrum* (Pep-H: Pepper Hexane, and Pep-EA: Pepper Ethylacetate) extracts were recorded to identify their bioactive compounds. The GC–MS chromatogram of Pep-EA was much clearer and a single major peak (52.4%) of piperidine, 1-(5-(1,3-benzodioxol-5-yl)-1-oxo-2,4-pentadienyl)-, and (Z,Z)- {syn. chavicine} was recorded (Figure 1A). Chavicine is one of the four geometrical isomers of piperine [22] and has been reported to enhance memory in the mice model [23,24].

In the case of Pep-H, a total of 29 peaks were registered with only 9 peaks over 5%, namely, Caryophyllene (5.79%), 2,4-Decadienamide, N-isobutyl-, (E,E)- {syn. pellitorine} (7.75%), (E)-9-Octadecenoic acid ethyl ester (5.65%) {syn. ethyl elaidate}, (E)-5-(Benzo[d][1,3]dioxol-5-yl)-1-(piperidine) {syn. dihydropiperine} (9.59%), (2E,4E,10E)-N-Isobutylhexadeca-2,4,10-trienamide {syn. pipercide} (11.40%), Piperidine, 1-(5-(1,3-benzodioxol-5-yl)-1-oxo-2,4-pentadienyl)-, (Z,Z)- {syn. chavicine} (8.71%), (E)-7-(Benzo[d][1,3]dioxol-5-yl)-1-(piperidine-1-yl)hept-6-en-1-one) {syn. piperolein A} (6.94%), (2E,4E,8E)-9-(Benzo[d][1,3]dioxol-5-yl)-1-(piperidin-1-yl)nona-2,4,8-trien-1-one) {syn. dehydropipernonaline} (7.69%), and (E)-9-(Benzo[d][1,3]dioxol-5-yl)-1-(piperidin-1-yl)hept-6-en-1-one) {syn. piperolein B} (10.03%) (Figure 1B).

The key bicyclic sesquiterpene contributing to the piquancy of Pep-H is β-Caryophyllene (BCP), which is also the first “dietary cannabinoid” with GRAS (generally recognized as safe) status and certified for food use by the FDA [25]. Apart from having a therapeutic role in several pathological conditions, it also has a positive impact on improving neurodegenerative diseases [26]. Pellitorine (PT), an amide alkaloid, has been reported for anti-septic, antibacterial, insecticidal, and anticancer activities [27,28] and also acts as a transient receptor potential cation channel, subfamily V, member 1 (TRPV1) antagonist, inhibiting exovanilloid-induced pain [29]. Dihydropiperine has a γ-aminobutyric acid (GABAA) receptor binding affinity. Piperolein A and B are known to activate thermosensitive receptors (TRP), TRPV1, and the transient receptor potential cation channel, subfamily A, member 1 (TRPA1), suggesting a role in thermoregulation [30]. In addition, pipercide exhibited anti-malarial properties [24,31] whereas piperine and dehydropiperonaline from *P. retrofractum* acted as anti-obesity agents [32].

### 3.2. Phytochemical Estimation and Antioxidant Potential of Piper nigrum Extract

The total phenolic content (TPC) and flavonoid content (TFC) were estimated in the extracts using colorimetric assays. Phenols and flavonoids are secondary metabolites with an important role in the growth, communication, and defense of plants. Pep-EA had a higher phenol and flavonoid content compared to Pep-H. The phenolic content in the Pep-H and Pep-EA extracts was calculated to be 14.78 ± 1.99 mg GAE/g and 24.10 ± 0.67 mg GAE/g, respectively, while the flavonoid content was 20.15 ± 0.78 mg QE/g (Pep-H) and 41.86 ± 0.69 mg QE/g (Pep-EA).

Previously, the methanolic extract of black pepper had a TPC of 6.71 ± 0.34 mg GAE/g and TFC of 63.11 ± 3.16 mg QE/g [33]. However, the TPC (22.69 ± 0.58 μg GAE/g) and TFC (3.65 ± 0.62 μg GAE/g) were quite low in the ethyl acetate extract [34].

The antioxidant potential of the extracts was also evaluated by different assays. The percent of radical scavenging activity observed in the DPPH assay was lower in the case of Pep-H (34.39 ± 0.24%) compared to Pep-EA (54.8 ± 0.39%). However, the ABTS^+^ radical scavenging activity and electron transfer (FRAP) were better in the case of Pep-EA (22.91 ± 1.19% and 41.65 ± 0.53 µM Fe^2+/^g) compared to Pep-H (14.79 ± 0.58% and 6.41 ± 0.01 µM Fe^2+/^g) at 50 μg/mL. In a previous study, the water extract of black pepper exhibited 26.67% (DPPH assay), 74.87% (ABTS assay), and 20.42% (FRAP assay) activity at 500 μg/mL [35]. The hydroalcoholic extract exhibited 43.1% (ABTS) and 43% (DPPH) activity [36]. The loss of phenols has been reported during the ripening and drying of black pepper, resulting in lower antioxidant activity compared to its green stage [37]. The higher TPC and TFC content in the case of Pep-EA can be correlated to the better antioxidant potential of Pep-EA; a positive correlation between the antioxidant potential and TPC has been reported earlier [38]. In GC–MS, chavicine (the most abundant isomer of piperine) was reported as the main phytocompound (52.4%) of Pep-EA. The antioxidant activity of piperine is well-established in vitro and in vivo studies [39,40]. However, substantial information on the antioxidant property of chavicine is lacking. From the antioxidant assay results obtained in our study, Pep-EA displayed better activity compared to Pep-H, suggesting the antioxidant potential of chavicine.

### 3.3. In Vitro Anti-Glycation Activity

Glycation is a non-enzymatic reaction that results in the formation of AGEs which are the cross-linked structures formed between proteins and reducing sugars, eventually leading to inflammation and oxidative stress in addition to affecting cell signaling. Chronic stress accelerates the formation and aggregation of AGEs in the body, which in turn fuels oxidative stress [41]. The AGEs are involved in the pathogenesis of age-related NDs, diabetic complications, chronic kidney disease, etc. [42]. Therefore, glycation is also an important therapeutic target for the treatment of NDs.

**Figure 2:**
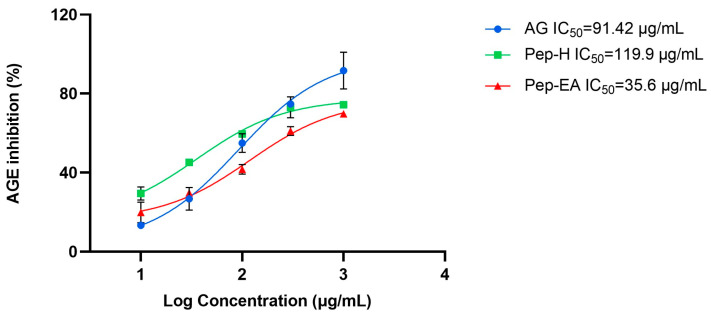
The anti-glycation activity exhibited by *Piper nigrum* extracts. Data are the mean ± SD of triplicates. Results were calculated using GraphPad Prism 9.5. Abbreviations: AG: Aminoguanidine; Pep-H: Pepper-Hexane; and Pep-EA: Pepper-Ethyl acetate.

The BSA-AGE fluorescence assay was used to assess the in vitro anti-glycation potential of varying concentrations of Pep-H and Pep-EA. After two weeks of incubation with the BSA-glucose buffer, the extent of glycation inhibition was calculated. Pep-EA potentially inhibited the glycation with an IC_50_ value of 35.6 μg/mL, much lower than Aminoguanidine, the positive control (IC_50_ 91.42 μg/mL). In a previous report, a similar IC_50_ value of 91.2 μg/mL was reported for Aminoguanidine [43]. Pep-H also exhibited similar anti-glycation activity (IC_50_ 119.9 μg/mL) as the control (Figure 2). Previously, black pepper (hydro-alcoholic) extracts inhibited AGE formation by 67% in the BSA-glucose model [36].

It has been known that spices are a rich source of polyphenols and could inhibit AGE formation considerably [44] through their antioxidant nature, protein interaction, metal chelating action, and by blocking the AGE receptor (RAGE) [45]. The strong anti-glycation potential of flavonoids is due to their binding to proteins, which might prevent AGE formation [46,47]. Previously, the antioxidant properties of piperine were reported for its in vitro and in vivo dose-dependent anti-glycation action [48,49]. Therefore, the lower IC_50_ value shown by Pep-EA compared to Pep-H can be correlated to its higher flavonoid content, better antioxidant profile, and higher chavicine (an isomer of piperine) content. In addition, piperine forms a stable albumin-piperine complex by interacting with its subdomain IIA, which could be another possible mechanism for the anti-glycation action [50]. It is speculated that chavicine might form a complex with the albumin, such as piperine, and inhibit glycation. The potent anti-glycation activity shown by Pep-H could be the result of the synergistic action of phytocompounds. The superior anti-glycation activity of Pep-EA makes it a prospective compound in therapeutics for the future development of novel anti-AGE inhibitors.

### 3.4. Acetylcholinesterase Inhibitory Activity

**Figure 3:**
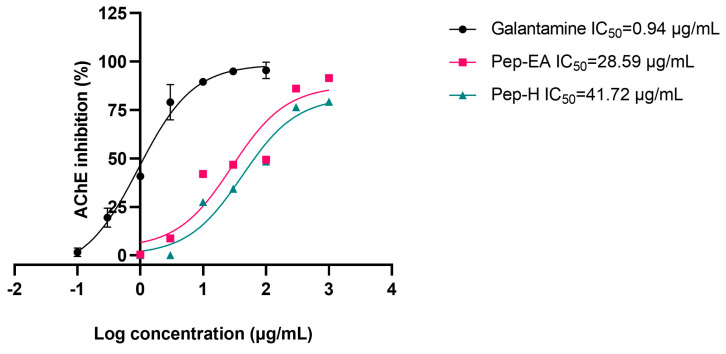
IC_50_ curve of *Piper nigrum* extracts with inhibitor control (Galantamine) against AChE. Data are the mean ± SD of triplicates. The IC_50_ values were calculated using GraphPad Prism 9.5. Abbreviations: Pep-H: Pepper-Hexane; Pep-EA: Pepper-Ethyl acetate.

To evaluate the neuroprotective effect of black pepper extracts, we also studied their anti-acetylcholine esterase (AChE; E.C.3.1.1.7) activity. The higher AChE induces apoptosis and affects synaptic integrity neurodevelopment [51] therefore AChE inhibition is desirable in the management of AD to maintain the level of the neurotransmitter acetylcholine (ACh) for cholinergic transmission. In this study, the pepper extracts displayed ~40% inhibition in preliminary screening at 100 μg/mL. Hence, the experiment to calculate IC_50_ values (half maximal inhibitory concentration) was conducted. The IC_50_ value of Pep-H was 41.72 μg/mL and that of Pep-EA was 28.59 μg/mL. In previous studies, the methanolic pepper extract had shown an IC_50_ value of 11.13 μg/mL [52] whereas the black pepper oil exhibited a strong AChE inhibition with a low IC_50_ value (5.9 μg/mL) [53]. The IC_50_ value of the positive control, Galantamine, was calculated as 0.94 μg/mL, like the previously reported value of 1.45 μg/mL [54]. The IC_50_ values of the extracts and the positive control have been shown in Figure 3.

**Figure 4:**
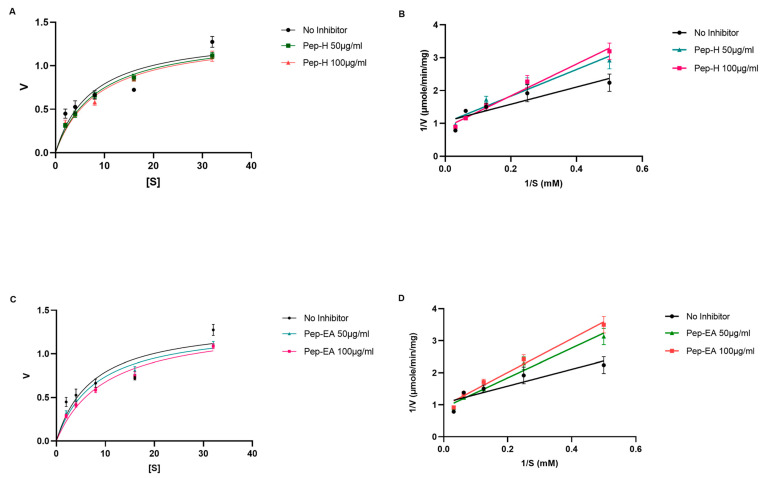
Michaelis-Menten (**A**,**C**) and Lineweaver-Burk (**B**,**D**) plot of AChE in the presence of 50 μg/mL and 100 μg/mL of *Piper nigrum* extracts (Pep-H and Pep-EA). The graphs were plotted using GraphPad Prism 9.5. Abbreviations: Pep-H: Pepper-Hexane; Pep-EA: Pepper-Ethyl acetate; V: Velocity of enzyme-catalyzed reaction; Vmax: Maximum velocity; and S: Substrate.

The Lineweaver-Burk plot was used to elucidate the mechanism of AChE inhibition. Competitive inhibition was observed for both Pep-H and Pep-EA (Figure 4) and the kinetic parameter values (Table 1) were calculated from a non-linear fit (Michaelis-Menten equation).

Similar Vmax (the maximum rate at which an enzyme is catalyzed when the enzyme is saturated by the substrate) values were obtained for the no-inhibitor (1.375 μmole/min/mg) with Pep-H (1.364 μmole/min/mg at 50 μg/mL and 1.372 μmole/min/mg 100 μg/mL) and Pep-EA (1.333 μmole/min/mg at 50 μg/mL and 1.360 μmole/min/mg 100 μg/mL), while an increase in the Km (the concentration of substrate which permits the enzyme to achieve half Vmax) values was observed: 7.37 mM (no-inhibitor), Pep-H (8.35 mM; 8.75 mM), and Pep-EA (8.05 mM;10.08 mM) at 50 μg/mL and 100 μg/mL, respectively (Figure 4). The increased Km value in the presence of an inhibitor reduces the affinity of the enzyme for the substrate. These results indicate a competitive inhibition pattern where the inhibitor competes with the substrate for binding to the active site of the enzyme.

An in silico study proposed that the presence of several functional groups (C=O, R–O–R, and C–OH) in the benzodioxol moiety of *P. nigrum* components (such as piperine), facilitate hydrophobic connections (five in the case of piperine) with amino acids present in the proteins, resulting in enhanced enzyme inhibition [8,55]. The molecular docking studies of *P. longum* extract using *T. californica* AChE suggested a possible hydrogen bonding with Tyr_70_ [56]. In a previous docking study, β-caryophyllene (a bio-active component of Pep-H) exhibited a lower binding affinity (−8.3 kcal/mol) and interacted with Tyr_114_, Trp_126_, Trp_351,_ and Phe_392_ of the *T*. *castaneum* AChE enzyme [57]. In addition, β-caryophyllene interacted with Phe_297_ and Trp_286_ residues in AChE from *Electrophorus* [58]. The molecular docking of some alkaloids from the ethanolic extract of *P. nigrum* also formed a hydrogen bond with Ser_200_ and His_440_ at the catalytic site of *T. californica* AChE [59]. The reason for a superior IC_50_ value of Pep-EA compared to Pep-H could be due to the higher content of chavicine compared to Pep-H. However, the Pep-H also displayed commendable AChE inhibition which could be the result of the synergistic action of bio-active components present in the extract as reported in the case of *P. longum* [56]. In the future, chavicine/piperine derivatives could be used as a pharmacophore for drug development in the treatment of NDs.

### 3.5. Piper nigrum Extract Reduced Aβ Fibrilization

**Figure 5:**
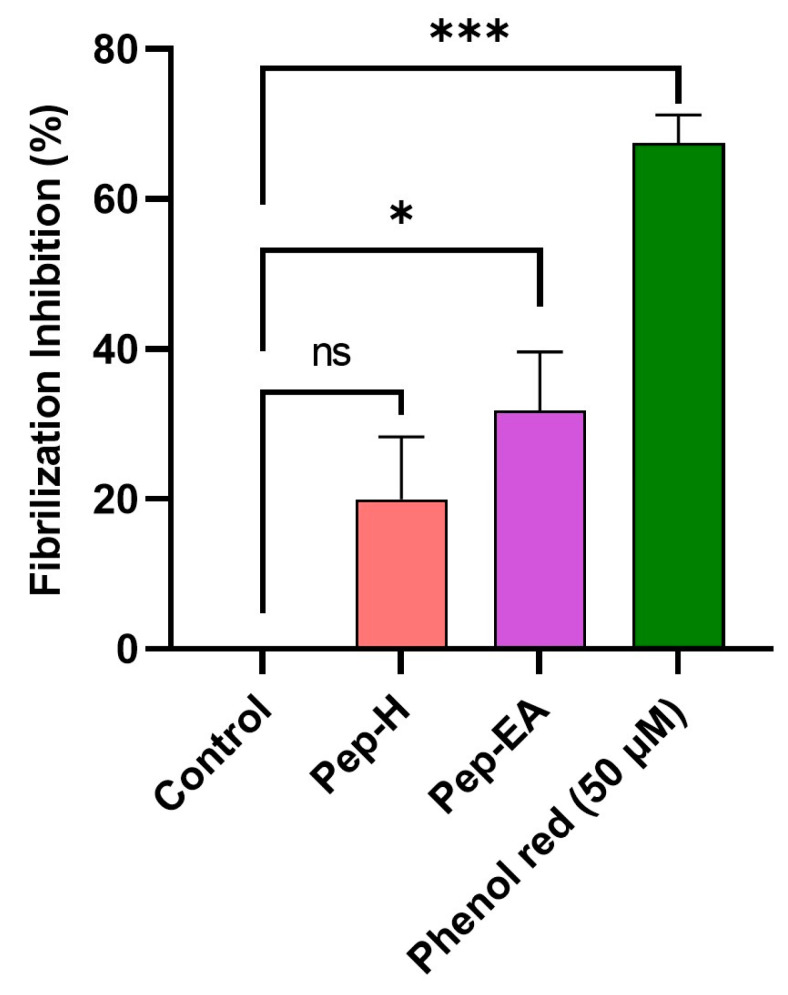
Aβ fibrilization inhibition in the presence of *Piper nigrum* extracts. The values are expressed as the mean ± SD (n = 3). Phenol Red (50 μM) was used as a positive control. A significant difference, * (*p* < 0.05) and *** (*p* < 0.001), using a one-way ANOVA followed by Dunnett’s post-hoc was observed in the reduction in fibrilization vs. the negative control (buffer + Aβ). Abbreviations: Pep-H: Pepper-Hexane; Pep-EA: Pepper-Ethyl acetate.

Aβ fibrilization inhibition activity of the pepper extracts was assessed using a ThT assay. The fluorescence signal is increased upon binding of ThT to the amyloid β-sheet. The extracts were screened at 100 μg/mL for Aβ fibrilization inhibition with Phenol red as a positive control. A statistically significant reduction was seen in the extracts compared to the control (buffer + Aβ). Phenol red exhibited a 67.49 ± 3.73% inhibition (*** *p* < 0.001) at 50 μM, comparable to the previously described value [60]. Pep-H was statistically non-significant while Pep-EA exerted statistically significant (* *p* < 0.05) inhibition of 31.84 ± 7.76% compared to the control (Figure 5).

Aβ fibrilization is the vital component of amyloid plaques in AD pathology which contribute to oxidative stress and neuroinflammation. It is speculated that the main component of Pep-EA, chavicine, might be interacting with the β sheet of the protein through π-stacking or hydrophobic interaction to exert anti-fibrilization activity [61]. Our results agree with a previous study, where *P. nigrum* (12.5 mg/kg/day) improved memory in an aluminum chloride-induced neurotoxicity mice model by significantly modulating the expression of amyloid-producing isoforms (APP770 and APP695) in the brain. The extract decreased the expression of the amyloidogenic APP770 isoform with a concomitant improvement in the expression of the APP695 (non-amyloidogenic) isoform in the hippocampus, amygdala, and cortex. Chavicine was identified as the main pharmacologically active component of the extract responsible for neuroprotection [23].

In a previous study, black pepper oil exhibited weak fibrilization inhibition (33.17 ± 6.67%) at 100 μg/mL [53]. However, no fibrilization inhibition was observed for black pepper water extract [62].

### 3.6. Cytotoxic Effect of Pepper Extracts on the SH-SY5Y Cell Line

**Figure 6:**
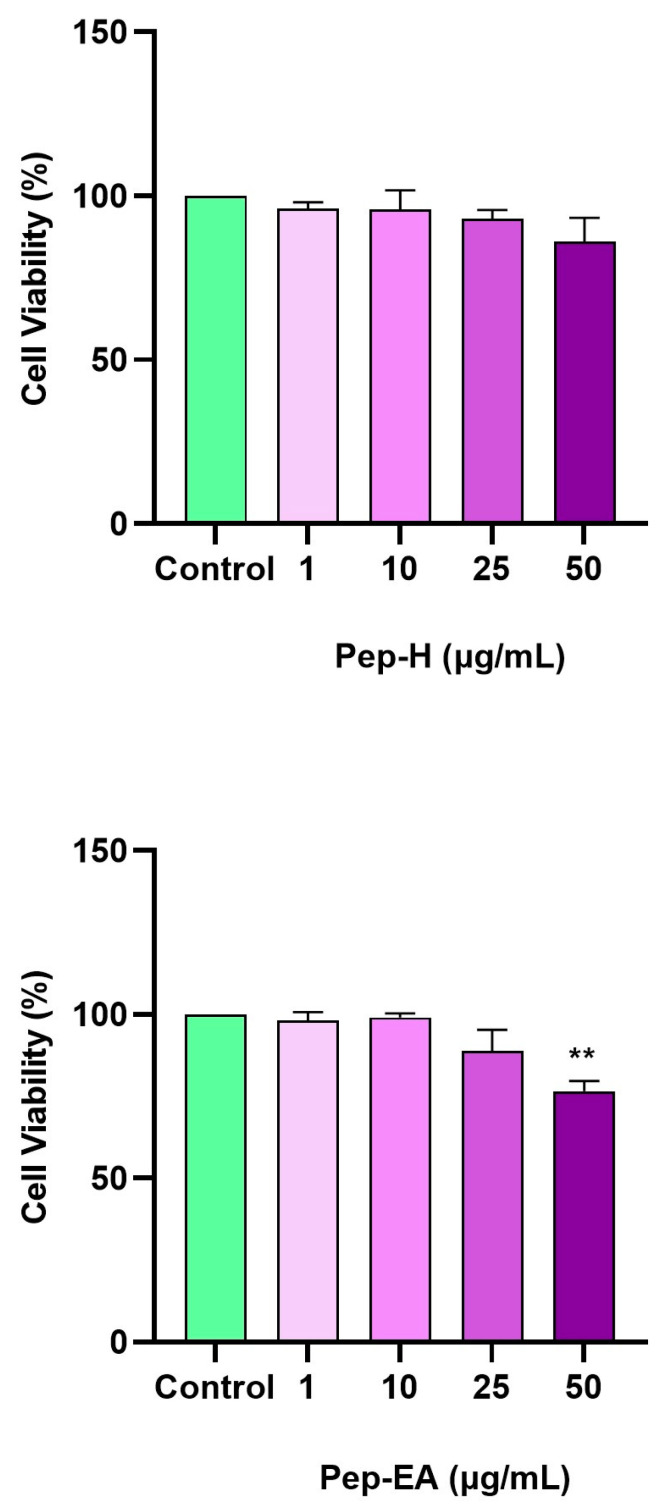
Cytotoxicity assay of *Piper nigrum* extracts on the SH-SY5Y cells. The cells were treated for 24 h with varying extract concentrations (1, 10, 25, and 50 μg/mL). The cell viability is reported as the percentage of the control group (100%). All data are presented as the mean ± SD (n = 3). A significant difference ** (*p* < 0.01) using a one-way ANOVA followed by Dunnett’s post-hoc was observed in the % cell viability vs. the control group (no treatment). Abbreviations: Pep-H: Pepper-Hexane; P-EA: Pepper-Ethyl acetate.

The cellular viability in the neuroblastoma cell line (SH-SY5Y) was analyzed using WST-8 dye after 24 h of treatment with different concentrations of the extracts (1, 10, 25, and 50 μg/mL). No cytotoxicity was observed up to 50 μg/mL for Pep-H, however, for Pep-EA, the cell viability decreased to 76.57% (** *p* < 0.01) at 50 μg/mL (Figure 6). Hence, to minimize cell death due to the extract toxicity, concentrations lower than 50 μg/mL were used for the subsequent cell-based assays.

### 3.7. Piper nigrum Provided Neuroprotection against H_2_O_2_-Induced Oxidative Stress in SH-SY5Y

The neuroprotective effects of the extracts were assessed by H_2_O_2_-induced oxidative stress in the SH-SY5Y cells. In the preliminary optimization experiment, H_2_O_2_ at 100 μM resulted in 50% cell survival after 6 h treatment. Therefore, 100 μM H_2_O_2_ was used to induce oxidative stress in SH-SY5Y cells pre-treated with different concentrations of the extracts (01, 0.3, 1, 3, 10, and 30 μg/mL) for 12 h.

**Figure 7:**
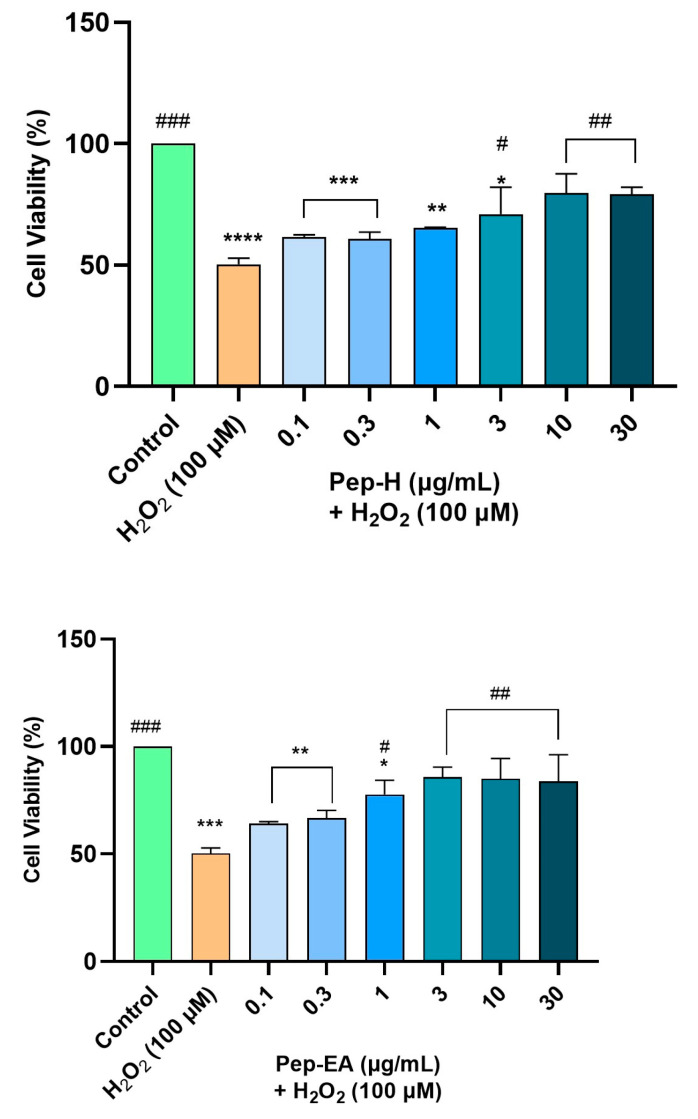
Neuroprotective effect of *Piper nigrum* extracts in H_2_O_2_-induced oxidative stress in neuroblastoma SH-SY5Y cells. The SH-SY5Y cells were preincubated with the extracts (0.1, 0.3, 1, 3, 10, and 30 μg/mL) for 12 h followed by 6 h of H_2_O_2_ (100 μM) treatment. The results indicate the % cell viability vs. the control cells, the mean ± SD (n = 3). A significant difference, */# (*p* < 0.05), **/## (*p* < 0.01), ***/### (*p* < 0.001), and **** (*p* < 0.0001) using a one-way ANOVA followed by Dunnett’s post-hoc test was observed in the % cell viability vs. untreated cells (*) and H_2_O_2_ treated cells (#). Abbreviations: Pep-H: Pepper-Hexane; Pep-EA: Pepper-Ethyl acetate.

Both the extracts displayed dose-dependent neuroprotection with a statistically significant effect at higher concentrations. Pep-EA performed better in protecting the cells against oxidative damage and significantly (# *p* < 0.05) increased the cell viability at 1 μg/mL and ## *p* < 0.01 at 3–30 μg/mL compared to H_2_O_2_ control. Pep-H significantly increased the cell viability at 3 μg/mL (# *p* < 0.05) and at 10 and 30 μg/mL (## *p* < 0.01) as compared to the H_2_O_2_ control. However, the lower concentrations (0.1–1 μg/mL) were ineffective for Pep-H (Figure 7). From the results, it can be concluded that 10 μg/mL of both extracts is the best concentration to display maximum neuroprotection, after which the effect remains almost constant. The neuroprotective mechanism was further explored by measuring the ROS and the MMP.

### 3.8. Piper nigrum Ameliorated H_2_O_2_-Induced ROS Generation

**Figure 8:**
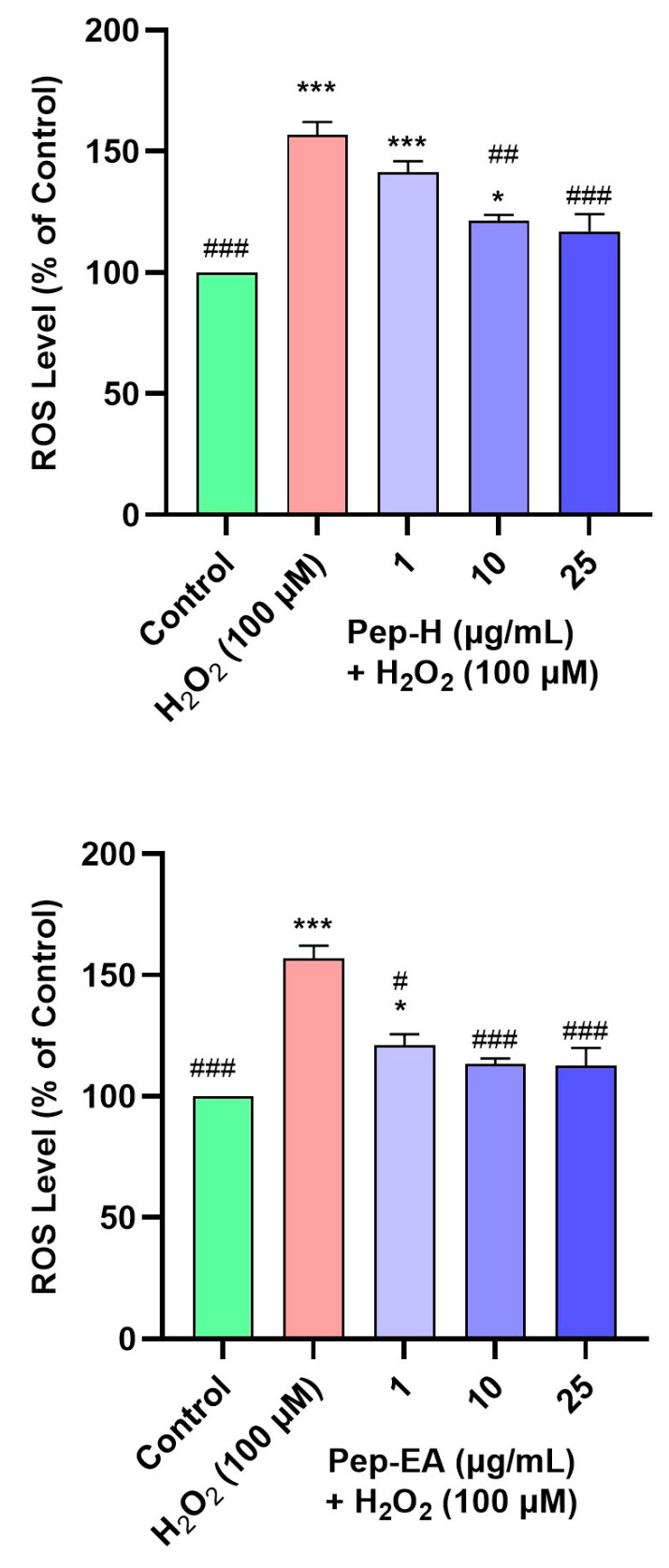
Effect of *Piper nigrum* extracts on H_2_O_2_-induced ROS production in SH-SY5Y cells. The SH-SY5Y cells were preincubated with the extracts (1, 10, and 25 μg/mL) for 12 h followed by 4 h of H_2_O_2_ (100 μM) treatment. The results indicate the % ROS level vs. the control cells (untreated cells). Values are the mean ± SD (n = 3). The data were analyzed by a one-way ANOVA followed by Dunnett’s post-hoc test. A significant difference, */# (*p* <0.05), ## (*p* <0.01), and ***/### (*p* <0.001), was observed in the % ROS vs. untreated cells (*) and H_2_O_2_ treated cells (#). Abbreviations: Pep-H: Pepper-Hexane; Pep-EA: Pepper-Ethyl acetate.

Oxidative stress and ROS generation are key characteristics of neurodegenerative diseases and have a damaging effect on cellular components including proteins, lipids, and DNA [63]. Hence, it is worth finding the compounds that can trim down intracellular ROS. As H_2_O_2_ is an important ROS generator, we used it to induce oxidative stress in the SH-SY5Y cell line. To evaluate the ROS scavenging activity of the pepper extracts, the cells were pre-treated with varying concentrations of the extracts for 12 h followed by H_2_O_2_ (100 μM) exposure for 4 h. The fluorescent dye (H2DCFDA) was used to monitor ROS production. H_2_O_2_ treatment generated 156.9 ± 5.0% ROS compared to the untreated cells (100%). Pre-treatment of the cells with extracts resulted in a dose-dependent decrease in ROS production in Pep-H. The Pep-EA was slightly more effective and significant at 1 μg/mL (121 ± 4.5%; *p* < 0.05) and 10 μg/mL (113.1 ± 2.4%; *p* < 0.001) compared to Pep-H (Figure 8). Thereby, 10 μg/mL of Pep-EA and 25 μg/mL of Pep-H are required for the maximum ROS reduction in the SH-SY5Y cell line. The dose-dependent reduction in ROS observed in our study indicates the antioxidant nature of the extracts which maintained a high level of cellular communication [64] and exerted a neuroprotective effect. Additionally, the effectiveness of Pep-EA in reducing ROS could be linked to better antioxidant activity and a higher chavicine content.

### 3.9. Piper nigrum Improved Mitochondrial Membrane Potential

Mitochondria are the major site of ROS generation during electron transport and stimulate the production of proinflammatory cytokines. The mitochondrial membrane potential (∆Ψm) is disturbed due to oxidative damage to the cell which affects membrane permeability and the release of Cytochrome C and/or pro-apoptotic factors in the cytoplasm. Therefore, the loss of MMP is regarded as an early marker of apoptosis and a major contributor to NDs [65,66].

**Figure 9:**
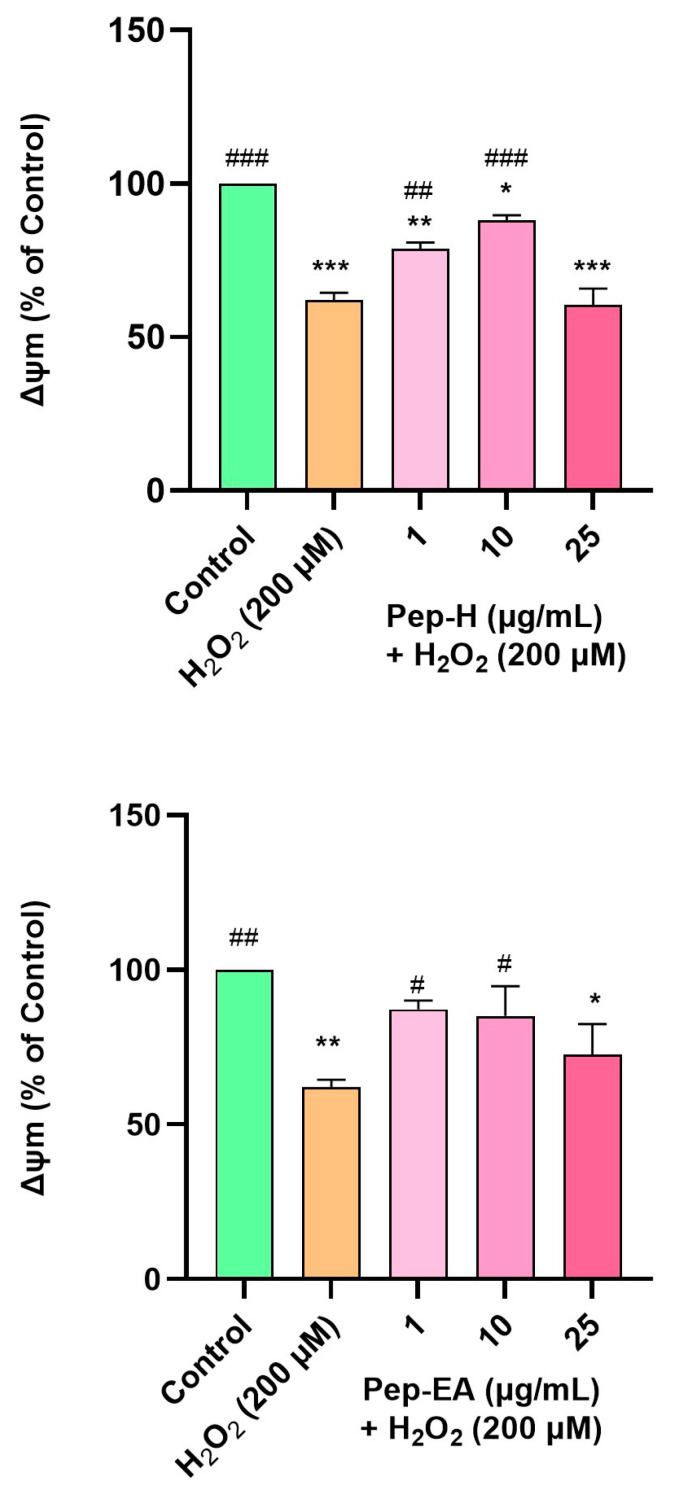
Mitochondrial membrane potential in SH-SY5Y cells exposed to 200 μM H_2_O_2_ for 2 h after 12 h pre-treatment with pepper extracts (1, 10, and 25 μg/mL). The results indicate % ∆Ψm vs. the control cells (untreated cells). Values are the mean ± SD (n = 3). The data were analyzed by a one-way ANOVA followed by Dunnett’s post-hoc test. A significant difference, */# (*p* < 0.05), **/## (*p* < 0.01), and ***/### (*p* < 0.001), was observed in the % cell viability vs. untreated cells (*) and H_2_O_2_ treated cells (#). Abbreviations: Pep-H: Pepper-Hexane; Pep-EA: Pepper-Ethyl acetate, and ∆Ψm: Mitochondrial membrane potential.

In the present study, H_2_O_2_ concentration and time of induction were optimized using a fluorescent dye (TMRE) which has an affinity for active mitochondria. In our experiment, H_2_O_2_ at 200 μM concentration reduced the MMP to ~50% of the untreated cells, hence, this condition was used for further examination. H_2_O_2_ treatment depolarized the mitochondria resulting in a decreased membrane potential. The cells pre-treated with Pep-H for 12 h followed by H_2_O_2_ (200 μM) treatment for 2 h (after removing the extracts) displayed a significant dose-dependent increase in MMP at 1 μg/mL (78.7 ± 2.0%; ## *p* < 0.01) and 10 μg/mL (88.0 ± 1.6%; ### *p* < 0.001), after which it declined significantly (60.5 ± 5.3%; *** *p* < 0.001). The effect of Pep-EA was milder than Pep-H, showing improvement in MMP at 1 μg/mL (87.1 ± 2.9%; # *p* < 0.05) and 10 μg/mL (85.0 ± 9.6%; # *p* < 0.05) which decreased afterward (72.5 ± 9.8%; * *p* < 0.01) at 25 μg/mL (Figure 9). It appears that Pep-H displayed neuroprotection by improving MMP and inhibiting Cytochrome C release into the cytosol. However, the decrease in ∆Ψm at higher concentrations (25 μg/mL) depicts the toxicity or ineffectiveness of both extracts in restoring MMP. In our experiment, Pep-EA effectively reduced ROS (Figure 8) but was not as effective in restoring MMP (Figure 9). No doubt mitochondria are the chief generators of intracellular ROS, however, they are not the only source. Other cellular sources include enzymes (xanthine oxidase, lipoxygenase, cyclooxygenase, and NADH/NADPH oxidase); the peroxisomal β-oxidation of fatty acids and microsomal metabolism of xenobiotics, etc. also contribute to ROS production [67,68]. Hence, it appears that Pep-EA is capable of scavenging ROS generated from other cellular sites as well.

Previously, the in vivo neuroprotective mechanism of piperine included protecting mitochondrial integrity via reducing oxidative stress and improving mitochondrial membrane potential and neuronal survival in a cerebral ischemia rat model [69,70] and streptozotocin-induced cognitively impaired rats [71].

## 4. Conclusions

The present study was conducted to explore the neuroprotective mechanism exerted by *Piper nigrum* extracts. H_2_O_2_, being the important mediator of oxidative stress, which eventually leads to Aβ aggregation, neuronal death, and neuroinflammation, was used to generate ROS in the human neuroblastoma SH-SY5Y. The *P. nigrum* extracts protected the cells from oxidative damage by reducing ROS production and maintaining mitochondrial membrane integrity, reflecting the antioxidant potential of the extracts. Pep-EA was able to scavenge ROS from other cellular sources as well. Additionally, the extracts displayed strong anti-glycation activity which might be possible due to the interaction of flavonoids with the proteins, preventing AGE formation. The extracts also competitively inhibited AChE and exhibited promising IC_50_ values, indicating that the bioactive components interacted with the amino acid residues at the active site of AChE. Moreover, black pepper-ethyl acetate extract significantly inhibited Aβ fibrilization. Better neuroprotection by Pep-EA is linked to a higher chavicine content. Hence, the multitarget neuroprotective mechanism presented by *Piper nigrum* makes it suitable for drug development in NDs. Previous studies have demonstrated that piperine (an isomer of chavicine) has a favorable pharmacokinetics profile with a high affinity towards brain tissue (98.4–98.5%), plasma protein (96.2–97.8%), and a brain distribution volume of 36.32 ± 1.40 mL/g [72]. Additionally, after dosing (100 and 200 mg), its C_max_ (the maximum drug concentration observed in the sampled blood or plasma) was reported as 3.77 μg/mL and 6.59 μg/mL, respectively, in healthy volunteers [73]. In contrast, the related literature on chavicine is limited; therefore, to fill this information gap, extensive investigations on the toxicity, bioavailability, and neuroprotective potential of chavicine in animal models and clinical trials are of utmost importance.

## Figures and Tables

**Table 1:** Kinetic parameters.

	Vmax (μmole/min/mg)	Km (mM)	Type of Inhibition
No Inhibitor	1.375	7.371	
Pep-H (50 μg/mL)	1.396	8.379	Competitive
Pep-H (100 μg/mL)	1.364	8.755	Competitive
Pep-EA (50 μg/mL)	1.333	8.052	Competitive
Pep-EA (100 μg/mL)	1.360	10.080	Competitive

## Data Availability

All data are provided in the article.
